# Reproductive coercion and violence against women with unplanned pregnancies in Turkiye

**DOI:** 10.1007/s00737-025-01668-w

**Published:** 2026-01-10

**Authors:** Ruşen Öztürk, Özlem Güner, Elmin Emi̇nov

**Affiliations:** 1 Faculty of Nursing Women Health and Disease Nursing Department, Ege University, İzmir, Turkey; 2 Present Address: Faculty of Health Sciences, Midwifery Department, Sinop University, Sinop, Turkey; 3 Gynecology and Obstetrics Department, Düzce University, Düzce, Turkey

**Keywords:** Abuse, Reproductive coercion, Unplanned pregnancy, Violence

## Abstract

**Purpose:**

This study aimed to examine the effects of reproductive coercion and violence on women with unplanned pregnancies in Türkiye.

**Methods:**

This descriptive and cross-sectional study was conducted between 2021 and 2022 with women who applied to the obstetrics and gynecology outpatient clinic. Data were collected using the “Descriptive Information Form,” the “Women Abuse Screening Tool (WAST),” and the “Reproductive Coercion Scale (RCS).

**Results:**

A total of 380 women were included in the study. It was found that 18.7% of women had experienced reproductive coercion, and 17.1% had been exposed to violence. A significant difference was observed between scores on the Reproductive Coercion Scale and the Violence Scale (*X*^2^ = 25.173, *p* < 0.001). Spouse’s alcohol consumption, reproductive coercion, residence, marital status, type of marriage, and pregnancy status together explained 27.8% of the variance in participants’ levels of violence.

**Conclusion:**

The study demonstrated that women in Türkiye experienced reproductive coercion during their reproductive years and that there was an association between violence against women and reproductive coercion. Notably, reproductive coercion and violence appeared to be significantly influenced by regional and cultural factors, reflecting variations observed across different countries and societies.

## Highlights

¬ Violence against women is a major public health issue.

¬ The literature highlights that women who are exposed to violence may also experience reproductive coercion.

¬ The results indicated a significant relationship between violence against women and reproductive coercion.

¬ Reproductive coercion and violence were significantly influenced by regional and cultural differences.

## Introduction

Violence against women has been recognized as a significant public health issue in Türkiye and worldwide (Hacettepe University Institute of Population Studies 2015; WHO 2018). It is widely regarded as a serious and widespread violation of women’s lives, health, and rights. According to the World Health Organization’s (WHO) 2018 report, approximately 31% of women globally have experienced either physical and/or sexual intimate partner violence or non-partner sexual violence in their lifetime. In Türkiye, national data from 2015 reported a violence rate of 38% among women, while the WHO reported a global average of 32% (Hacettepe University Institute of Population Studies 2015; WHO, 2021). The consequences of violence against women extend beyond physical harm and significantly affect reproductive health. Negative outcomes include unplanned pregnancies, poor maternal outcomes, sexually transmitted infections, and limited access to contraceptive and healthcare services, often because of victimization through physical or sexual partner violence (Miller and Silverman 2010). Consistent evidence has shown that exposure to intimate partner violence (IPV) is associated with an increased risk of unplanned pregnancy (Acharya et al. 2019; Ahinkorah et al. 2020; Park et al. 2016; Williams et al. 2008).

Unplanned pregnancies are defined as those occuring when a woman does not wish to become pregnant at the time of conception, even if the pregnancy might be welcomed later. In contrast, untimely pregnancies occur at inappropriate or unexpected times (CDC, 2015). Previous study has suggested a complex and multifactorial relationship between unplanned pregnancies, IPV, and abortion. Contributing factors include non-consensual sexual intercourse, fear of negotiating condom or contraceptive use, inconsistent contraceptive practices, and partner interference with healthcare access (Miller and Silverman 2010). Multicounty analyses have demonstrated that experiencing IPV increased women’s risk of unplanned pregnancy by approximately 13% to 30%, with sexual violence increasing this likelihood by 1.4 to 2.3 times (Maxwell et al. 2018; Acharya et al. 2019; Ahinkorah et al. 2020; Ajayi et al., 2020; McCloskey 2016). In Bangladesh, women with unplanned pregnancies were more likely to experience both physical and sexual violence (Kamal 2013).

In Türkiye, 15% of births have been reported as unwanted, 11% of pregnancies unplanned, and 15% of women as having had at least one induced abortion. Furthermore, 12% of women reported an unmet need for family planning (TNSA 2018). According to the CISU platform (2021), the rate of unwanted pregnancies ranged between 15% and 46.2%, and 45.8% of these pregnancies occuring without the use of any contraceptive method (Esin et al. 2021).

A critical mechanism underlying the link between violence and reproductive outcomes is reproductive coercion (RC). RC is a distinct form of intimate partner violence characterized by behaviors that interfere with a woman’s autonomy over reproductive decision-making, such as contraceptive sabotage, pregnancy pressure, or coercion regarding continuation or termination of pregnancy (Miller et al. 2010; Brandi, 2018; Fay 2018). While IPV encompasses a wide range of physical, emotional, and sexual abuse, RC specifically targets reproductive control and often operates through subtle or manipulative strategies (Grace and Anderson 2018; Tomar et al., 2020).

Reproductive coercion, manifested through contraception sabotage, verbal pressure, threats, or forced control over pregnancy outcomes (continuation or termination), has been associated with unintended pregnancies, adverse health consequences, sexually transmitted infections, and increased exposure to violence (Fay 2018; Brandi 2018; Miller 2010). Therefore, when physical and sexual violence co-occur, the likelihood of reproductive coercion nearly doubles (Miller et al. 2010). Recent evidence further indicates that these associations are closely linked to reproductive control, defined as attempts by male partners to restrict or manipulate a woman’s reproductive choices (Kirk and Miller 2014).

Women’s experiences of reproductive coercion and abuse vary significantly across different national, cultural, and socio-legal contexts (Grace & Faming, 2016). In Cote d’Ivoire, ethnicity and religion were identified as influential factors, with women from the Guere ethnic group or those practicing traditional religions more likely to report coercion by in-laws (Gupta et al. 2012). In the United States, Nikolajski et al. (2015) found that nearly 40% of participants reported reproductive coercion, with African American women disproportionately affected. These narratives highlighted broader structural issues such as mass incarceration and social instability in low-income African American communities. Similarly, research in Australia showed that women identifying as Aboriginal or culturally and linguistically diverse were more likely to report reproductive coercion (Price et al., 2022). Taken together, these findings suggest that reproductive coercion is shaped not only by cultural and ethnic structures but also by the intersection of social, structural, socioeconomic, and gender-based power dynamics.

The concept of reproductive coercion (RC) has been developed within an international framework emphasizing power, gendered control, and structural inequalities (Grace and Anderson 2018). Broader theoretical models, such as Gender and Power Theory (Connell 1987) and the Ecological Model of Violence (Bronfenbrenner 1979), also provide valuable perspectives for understanding IPV and reproductive coercion. Gender and Power Theory highlights how societal gender norms and patriarchal dynamics shape intimate relationships, limit women’s autonomy, and increase vulnerability to reproductive control. The Ecological Model of Violence offers a multi-layered lens to examine how individual, relational, community, and societal factors interact to perpetuate IPV and reproductive coercion. These frameworks are particularly relevant in the Turkish context, where socio-cultural norms, regional disparities, and gender roles strongly influence women’s reproductive rights and exposure to violence. Thus, this study aimed to enhance understanding of the dynamics of reproductive coercion among women in Türkiye and to provide insights into the broader socio-cultural and structural factors that shape women’s reproductive health experiences.

When reproductive coercion and physical or sexual violence co-occur, the risk of unintended pregnancy is significantly compounded (Miller et al. 2010; Kirk and Miller 2014; Thiel de Bocanegra, 2010). Despite its public health importance, RC often goes unrecognized by both women and healthcare providers due to its covert and complex nature (Park et al. 2016). Reproductive health providers are uniquely positioned to screen for RC and IPV and to provide early interventions to mitigate harm. Although many international studies have examined the links between IPV, RC, and unplanned pregnancy, there remains a lack of culturally contextualized research, especially in Turkiye. Given the high prevalence of gender-based violence in the country and the sociocultural barriers to reproductive autonomy, this study aimed to explore the impact of intimate partner violence and reproductive coercion on unplanned pregnancies among women of reproductive age in Türkiye. By addressing a gap in national data, this research sought to contribute to the literature and inform context-specific interventions.

## Method

This descriptive and cross-sectional study was conducted in Türkiye between 2021 and 2022 with women in their reproductive period who applied to the obstetrics and gynecology polyclinics (seeking various reproductive health services such as prenatal care, family planning, and routine gynecological examinations and screenings) of a university hospital and a state hospital located in the northern and eastern regions. Two regions were selected due to similarities in sociocultural characteristics, which supported representativeness and feasibility of data collection. The Western Black Sea region is characterized by a moderate violence rate and mid-level socioeconomic status, whereas Northeastern Anatolia has a higher prevalence of violence and lower socioeconomic indicators (Hacettepe University Institute of Population Studies 2015).

Random sampling was employed for sample selection. Each day, the list of women attending the clinics was reviewed, and among those who met the inclusion criteria, women whose protocol numbers ended with an odd digit (e.g., 1, 3, 5, 7, 9) were invited to participate. This method ensured a simple and objective randomization process. The sample size was calculated using the estimated prevalence of violence against women in Türkiye, reported as 32% by national and international sources (WHO, 2021). The calculation was performed using an online sample size calculator (http://www.istatistikakademisi.com/single-group-proportional.html), with a 95% confidence level and a 5% margin of error. Based on this analysis, the minimum required sample size was determined to be 334 women. To account for potential data loss and to increase statistical power, a total of 380 women were included in the study. Recruitment was carried out through face-to-face interviews in primary healthcare centers. Inclusion criteria were: being literate, having no hearing, vision, or comprehension difficulties, being in the reproductive period, and being married or having a partner who agreed to participate in the study.

## Data tools

Data were obtained using the “Descriptive Information Form,” “Women Abuse Screening,” and “Reproductive Coercion Scale.”

### Descriptive information form

This form consisted of 22 items, of which the first 10 covered socio-demographic characteristics (e.g., age, marital status, occupation, education), while the remaining 12 addressed obstetric history.

## Women abuse screening scale

The Women Abuse Screening Scale (WASS) was developed by Brown et al. (2000) to assess partner violence against women and was adapted into Turkish by Tatlıcalı (2009). The scale comprises of eight items and demonstrates high internal consistency (Cronbach’s α = 0.95), with the Turkish adaptation exhibiting a reliability coefficient of 0.81. The items are rated on a three-point Likert-type scale. Items 1 and 2 use the response options “very stressful,” “a little stressful,” and “not stressful,” while Items 3 to 8 use “often,” “sometimes,” and “never.” (A sample item is: “How would you describe your relationship in general?” with response options: (a) very stressful, (b) a little stressful, (c) not stressful; another sample item is: “Do arguments make you feel humiliated or bad?” with response options: (a) often, (b) sometimes, (c) never.). Each item is scored on a 3-point scale, resulting in a total score range from 0 to 16. While there is no universally fixed cut-off in the original scale, many studies use a cut-off score of 12 or higher to indicate the presence of violence.

## Reproductive coercion scale

The Reproductive Coercion Scale (RCS) is a measurement tool developed to assess behaviors by a partner or spouse that interfere with autonomous reproductive decision-making, such as pregnancy coercion and condom manipulation. The scale was originally developed by McCauley et al. (2017) to define and improve its psychometric properties for use in research and clinical applications. In 2021, the short form of the scale was adapted to Turkish culture by Öztürk and Güner. In a study involving 4,674 young women aged 16–29 years, reproductive pressure included pregnancy coercion and conscious manipulation of condoms to induce pregnancy. While the long form of the scale consisted of nine items, a short form of five items was created by McCauley to make it easier for clinicians to use. The short form is a unidimensional scale, assessing reproductive coercion over the past three months. The scale is administered using a dichotomous yes/no (1/0) response format. Cronbach’s α was 0.72. There is no established cut-off score for the scale. An increase in the total score indicates a higher level of reproductive coercion.

### Data analysis

Data were analyzed using SPSS version 25.0. Descriptive statistics (numbers, percentages, and means) were used to summarize the data. For group comparisons, parametric tests (Student’s *t* test, one-way ANOVA) were applied when assumptions were met. For data that did not meet the assumptions, nonparametric tests (Mann–Whitney *U* test and Kruskal–Wallis test) were used. Associations between categorical variables were examined with the chi-square test, and effect size was evaluated with the Phi/Cramér’s V coefficient. Predictors of violence and reproductive coercion were identified using linear regression analysis.

### Ethics statement

The study was approved by the Sinop University Human Research Ethics Committee (dated 10.04.2021, decision number 2021/44). Clinical trial registration was not required. All study materials (flyers and advertisements, informed consent forms, and safety protocols) were evaluated by the Sinop University Institutional Review Board. The informed consent procedure was obtained in accordance with institutional review board guidelines. The aim and scope of the study were clearly communicated to the participants. Written informed consent was obtained from all participants. Data were collected in private waiting rooms where men were not permitted, ensuring confidentiality and a safe environment.

## Results

A total of 380 women participants were invited to participate in the study, and all agreed to take part voluntarily. Each women participant completed all three data collection forms in full. No incentives or payments were provided to the women participants for their involvement in the study. The mean age of the women participants was 36.6 ± 10.2, and the mean age at first pregnancy was 22.75 ± 4.63 years. The mean number of pregnancies was 3.55 ± 2.52, and the mean of voluntary abortion was 1.27 ± 0.804. It was determined that 31.1% of the women participants had completed primary school, and 52% were married by an acquaintance (Table 1).

**Table 1:** The comparison of Socio-Demographic, Violence, coercion scale score mean of women

	*n*	%	WAST* X̄±sd	RCS* X̄±sd
Educational status				
Literate/illiterate Primary school High school Faculty/college/master	65 117 91 106	17.1 31.1 23.9 27.9	11.67 + 2.49 10.78 + 2.44 10.31 + 2.29 9.57 + 1.31	0.36 + 0.6 0.23 + 0.46 0.14 + 0.38 0.11 + 0.31
Test/p**			*F* = 12.373, ***p*** **< 0.001**	*F* = 5.408, ***p*** **= 0.01**
Age (yr)				
15–24 25–34 35–49	43 126 211	11.3 33.2 55.5	201.70 197.43 183.15	216.05 198.73 180.38
			*X*^2^ = 1.953, *p* = 0.377	*X*^2^ = 10.556, ***p*** **= 0.005**
Educational status of spouse				
Literate/illiterate Primary school High school Faculty/college/master	32 119 117 111	8.4 31.6 30.8 29.2	12.21 + 3.20 11.02 + 2.55 10.18 + 1.96 9.72 + 1.92	0.50 + 0.67 0.25 + 0.48 0.16 + 0.39 0.10 + 0.31
			*F* = 13.179, ***p*** **< 0.001**	*F* = 7.543,***p*** **= 0.001**
Marital status				
Married Single	362 18	95.3 4.7	186.45 261.25	190.71 186.25
			*U* = 1,966.500, ***p*** **= 0.004**	*U* = 3,181.500, *p* = 0.804
Type of marriage				
Companionate Arranged	198 182	52.0 48.0	9.97 + 2.16 11.04 + 2.48	0.17 + 0.39 0.23 + 0.19
			*t* = −4.468, ***p*** **< 0.001**	*t* = −1.389, *p* = 0.16
Working status				
Housewife Employee Retired	242 128 10	63.7 33.7 2.6	204.29 168.90 115.60	195.88 183.11 155.00
			*U* = 13.890,***p*** **= 0.001**	*X*^2^ = 4.824, *p* = 0.090
Residence				
Metropolis City District Village	43 217 50 70	11.3 57.1 13.2 18.4	183.57 181.37 202.84 211.85	168.08 191.61 178.95 209.07
			*X*^2^ = 5.083, *p* = 0.166	*X*^2^ = 9.555,***p*** **= 0.023**
Income status				
Less income Balance of income − expense Much more income	104 187 89	27.4 49.2 23.4	216.90 188.12 162.21	211.59 189.65 167.64
			*X*^2^ = 12.369, ***p*** **= 0.002**	*X*^2^ = 16.843,***p*** **< 0.001**
Drinking status				
No alcohol Social drinker Alcoholic	278 96 6	73.2 25.3 1.6	195.88 164.14 332.17	197.32 168.67 223.75
Total	**380**		*X*^2^ = 16.772,***p*** **< 0.001**	*X*^2^ = 11.842,***p*** **= 0.003**

Note.* WAST; Women Abuse Screening Tool. RCS; Reproductive Coercion Scale

**X^2^ = Kruskal-Wallis ranked one-way analysis of variance

*F* = One-way ANOVA

U = Mann-Whitney U

T = t test were used

Research findings showed that 18.7% of the women participants experienced reproductive coercion, and 17.1% were subjected to violence. In the 5-item measure of reproductive pressure, 14.5% (*n* = 54) of participants reported that their partner had told them not to use birth control methods (e.g., pills, IUD), and 3.8% (*n* = 14) indicated that they had been forced to have unprotected (condom-free) sexual intercourse in order to become pregnant. In the analysis, factors such as lower educational attainment of both the woman and her spouse, traditional marriage type, being a housewife, low socioeconomic status, and high levels of alcohol consumption were found to be significantly associated with increased risk of exposure to violence (*p* < 0.05) (Table 1). Violence scores differed significantly according to pregnancy status and the decisions regarding pregnancy continution(*p* < 0.05) (Table 2). Moreover, violence scores were negatively correlated with age at first pregnancy, number of pregnancies, and time elapsed since the last pregnancy (respectively *r*=−0.174, *p* < 0.001; *r*=−0.288, *p* < 0.001; *r*=−0.116, *p* < 0.001). In contrast, a significant positive correlation was found between violence scores and the number of births (*r* = 0.188, *p* < 0.001).It was determined that there was a significant difference in factors associated with reproductive coercion, such as women’s education level, spouse’s education level, age, place of residence, high income level, and high alcohol consumption (*p* < 0.05) (Table 1). Significant differences in reproductive coercion scores were observed concerning pregnancy status, continuation decision, non-use of contraception by the partner, and pregnancies that the woman solely desired (*p* < 0.05) (Table 2). Additionally, reproductive coercion scores showed a negative correlation with age at first pregnancy, number of pregnancies, and time elapsed since the last pregnancy (respectively *r*=−0.192, *p* < 0.001; *r*=−0.145, *p* < 0.001; *r*=−0.169, *p* < 0.001).

**Table 2:** The comparison of obstetric Features, violence and coercion scale score mean of women

	*n*	%	WAST* X̄±sd	RCS* X̄±sd
Pregnancy status				
Yes No	100 280	26.3 73.7	211.12 182.43	204.10 185.64
Test/p**			*U* = 11,838,,***p*** **= 0.022**	*U* = 12,640, ***p*** **= 0.033**
The desired status of previous and present pregnancy^a^				
Wanted pregnancy Those who are undesirable first but decide to continue the pregnancy Those who terminate or will terminate an unplanned pregnancy	188 120 31	55.5 35.4 9.2	9.95 + 2.04 11.49 + 2.51 11.15 + 3.02	0.17 + 0.42 0.30 + 051 0.12 + 0.33
			*F* = 16.904, ***p*< 0.001**	*X*^2^ = 3.649, ***p*** **= 0. 018**
Who decides to terminate				
Me Co-decision Using drugs during pregnancy Breast cancer	5 17 2 1	20.0 68.0 8.0 4.0	7.40 14.68 15.00 8.50	15.00 12.50 12.50 12.50
			*X*^2^ = 4.435, *p* = 0.218	*X*^2^ = 4.000, *p* = 0.261
Reason for unplanned pregnancy^b^				
Not using contraception methods Having problems with not using contraception methods Pregnancy despite using a method My spouse does not want to use a method My spouse only wants to use a method Not ejaculating inside	47 33 59 14 12 5		83.89 79.56 82.53 101.00 105.42 83.70	90.99 76.48 67.31 131.18 126.75 81.10
			*X*^2^ = 4.210, *p* = 0.520	*X*^2^ = 53.870, ***p*** **≤ 0.001**
Using a method right now^b^				
Yes No	145 135	38.2 35.3	145.21 134.37	145.47 134.08
			*U* = 8,960, *p* = 0.254	*U* = 8,922, *p* = 0.065

Note; WAST; Women Abuse Screening Tool. RCS; Reproductive Coercion Scale

**X^2^ = Kruskal-Wallis ranked one-way analysis of variance

*F* = One-way ANOVA

U = Mann-Whitney U

T = t test were used

^a^It was calculated out of 339 women who responded. More than one answer was given

^b^The pregnant group was excluded (*n* = 280)

This study demonstrated a significant association between women’s violence and reproductive coercion scales (*X*^2^ = 25.173, *p* < 0.001), with a moderate effect size (Phi = 0.366), indicating that women with a history of violence were significantly more likely to experience reproductive coercion. According to these data, 41.5% of women exposed to violence also experienced reproductive coercion. A significant difference was also observed between violence status and geographic region (χ²= 21.161, *p* < 0.001; Phi = 0.336), reflecting a moderate effect size. Participants residing in the eastern region experienced significantly higher levels of both violence and reproductive coercion. Furthermore, a significant association was found between reproductive coercion and region (*X*^2^ = 33.576, *p* < 0.001), with a moderate effect size (Phi = 0.397). Unplanned pregnancies showed significant differences with both reproductive coercion (χ² = 7.121, *p* = 0.028; Phi = 0.352) and violence (χ² = 23.569, *p* < 0.001; Phi = 0.363) scales. Both associations demonstrated moderate effect sizes, suggesting that unplanned pregnancies are meaningfully related to increased levels of both reproductive coercion and violence (Table 3).

**Table 3:** The comparison of Violence, coercion presence and unplanned pregnancy differences

	*n*	%	Test/*p*	Phi/Cramér’s V
Reproductive Coercion Scale Reproductive Coercion				
No Yes	309 71	81.3 18.7		
Women Abuse Screening Tool Violence Against Women				
No Yes	315 65	82.9 17.1		
Comparison of reproductive coercion with violence scale	**No**	**Yes**		
No violence Presence of violence	271 (87.7%) 38 (12.3%)	44 (62%) 27 (38%)	*X^2^ = 25.173 ***p*** **< 0.001**	0.366
Comparison of reproductive coercion with area	**North**	**East**		
No violence Presence of violence	183 (92.4%) 15 (7.6%)	126 (69.2%) 56 (30.8%)	*X*^2^ = 33.576 ***p*** **< 0.001**	0.397
Comparison of violence and area	**North**	**East**		
No violence Presence of violence	181 (91.4%) 17 (8.6%)	134 (73.6%) 48 (26.4%)	*X*^2^ = 21.161 ***p*** **< 0.001**	0.326
Comparison of pregnancy and coercion	**No**	**Yes**		
Wanted pregnancy Those who are undesirable but decide to continue the pregnancy Those who terminate or will terminate an unplanned pregnancy	159 87 27	29 33 5	*X*^2^ = 7.121 ***p*** **< 0.028**	0.352
Comparison of pregnancy and violence	**No**	**Yes**		
Wanted pregnancy Those who are undesirable first but decide to continue the pregnancy Those who terminate or will terminate an unplanned pregnancy	170 84 22	18 36 10	*X*^2^ = 23.569 ***p*** **< 0.001**	0.363

Note. The percentage of columns was given

*X^2^ = T he chi-square test was used, Phi/Cramér’s V has been used in calculating the effect size

The linear regression analysis revealed that spouse’s alcohol consumption (B = 0.563, 95% CI [0.183–0.762], *p* = 0.027), pregnancy status (B = 0.441, 95% CI [0.020–0.789], *p* = 0.041), reproductive coercion (B = 1.392, 95% CI [1.091–2.060], *p* < 0.001), marital status (B = 3.186, 95% CI [2.160–4.213], *p* < 0.001), type of marriage (B = 0.821, 95% CI [0.563–1.488], *p* = 0.001), and residence (B = 0.786, 95% CI [0.049–0.252], *p* = 0.009) significantly predicted women’s exposure to violence. Specifically, women whose spouses consume alcohol had violence scores 0.563 points higher, pregnant women had scores 0.441 points higher, and those experiencing reproductive coercion had scores 1.392 points higher, controlling for other factors. Women who were single had violence scores 3.186 points higher compared to married women, and those in arranged marriages had violence scores 0.821 points higher than those in companionate marriages. Additionally, women living in villages had violence scores 0.786 points higher than those living in urban areas. Overall, the model explained 27.8% of the variance in violence scores (R² = 0.278, F = 21.203, *p* < 0.001) (Table 4).

**Table 4:** Regression analysis results related to violence

Variables	B	S.H	β	t	*p*	VIF	95% CI
Stable	3.162	0.843		3.749	0.000		4.180–7.379
Drinking status for spouse	0.563	0.254	0.111	2.215	0.027	1.159	0.183–0.762
Pregnancy status	0.441	0.215	0.120	2.054	0.041	1.566	0.020–0.789
Reproductive coercion	1.392	0.261	0.262	5.342	0.000	1.102	1.091–2.060
Marital status	3.186	0.532	0.287	5.985	0.000	1.051	2.160–4.213
Type of marriage	0.821	0.238	0.169	3.452	0.001	1.100	0.563–1.488
Residence	0.786	0.298	0.162	2.637	0.009	1.723	0.049–0.252

Note. *R* = 0.527, *R*^2^ **=** 0.278, *F* = 21.203, *p* < 0.001, adjusted *r* = 0.265, Durbin–Watson statistic = 1.989

Linear regression analysis was conducted to identify predictors of reproductive coercion (Table 5). Spouse’s education level, residence, violence scores, and pregnancy status collectively explained 18.5% of the variance in reproductive coercion among participants (R² = 0.185, F = 18.988, *p* < 0.001). Since an increase in the total score indicates a higher level of reproductive coercion, the model showed that higher spouse education (B = −0.074, 95% CI [−0.098, −0.005], *p* = 0.002) and being pregnant (B = −0.157, 95% CI [−0.155, 0.039], *p* < 0.001) were associated with lower reproductive coercion scores, suggesting protective effects. In contrast, residence (B = 0.288, 95% CI [−0.023, 0.077], *p* < 0.001) and violence scores (B = 0.045, 95% CI [0.031, 0.069], *p* < 0.001) were positively associated with reproductive coercion, indicating that living in villages and experiencing higher levels of violence increase the risk of reproductive coercion. Although the confidence interval for residence included zero, the positive B value suggests a tendency toward higher coercion in rural areas. Specifically, women with more educated spouses had reproductive coercion scores 0.074 points lower, pregnant women had scores 0.157 points lower, women living in villages had scores 0.288 points higher, and women with higher violence scores had reproductive coercion scores 0.045 points higher, controlling for other factors.

**Table 5:** Linear regression analysis results related to reproductive coercion

Variables	B	S.H	β	t	*p*	VIF	95% CI
Stable	−0.180	0.164		−1.096	0.274	—	−0.482 - −0.307
Educational status for spouse	−0.074	0.023	−0.165	−3.169	0.002	1.115	−0.098- −0.005
Residence	0.288	0.055	0.315	5.238	0.000	1.485	−0.023–0.077
Pregnancy status	−0.157	0.042	−0.227	−3.724	0.000	1.520	−0.155–0.039
Violence	0.045	0.010	0.239	4.499	0.000	1.158	0.031–0.069

Note. *R* = 0.430, *R*^2^ = 0.185, *F* = 18.988, *p* < 0.001, adjusted *r* = 0.176, Durbin–Watson statistic = 1.725

## Discussion

In this study, 44.6% of the participating women experienced unplanned pregnancy. Rates of an unplanned pregnancies vary internationally, ranging from 15% to 46.2% in Türkiye, 27.9% in Iran, 27.1% in Ethiopia, 38.2% in Pakistan, 68% in Nepal, and 49% in Kenya (Esin et al. 2021; Habib, 2017; Ikamari et al. 2013; Jalali et al. 2019; Mohammed et al. 2016; Puri et al. 2016). Our findings are consistent with these national and international reports. Unplanned pregnancies pose substantial risks not only for reproductive health but also for maternal and public health (Hajizadeh and Nghiem 2020; Jalali et al. 2019; Vakili et al. 2011). According to the WHO (2019), 74 million women in low- and middle-income countries experience unplanned pregnancies annually, resulting in 25 million unsafe abortions and 47.000 maternal deaths. Preventing unplanned pregnancies and identifying their determinants are therefore critical priorities for health policy.

In the present study, 17.1% of women reported experiencing violence. Variables such as spouse’s alcohol consumption, reproductive coercion, residence, marital status, type of marriage, and pregnancy status significantly predicted exposure to violence, explaining 27.8% of the variance. Notably, reproductive coercion and marital status were the strongest risk factors, while residence, pregnancy status, and spouse’s alcohol consumption appeared as protective factors. According to WHO (2019), approximately one in three women worldwide experiences physical or sexual violence, and risk factors include young age, alcohol use, informal marriages, and history of abuse. Protective factors include higher socioeconomic status, education, and formal marriage. Studies in Türkiye similarly link intimate partner violence with socio-demographic variables such as education level, income, family structure, and marriage type (Altınay and Arat 2008; Efe and Ayaz 2010). Evidence from Ethiopia and Pakistan also points to associations between low education, alcohol use, and increased violence risk (Hindin et al. 2008; Nasrullah et al. 2009). Our data particularly underscore the heightened risk of violence among women with low education and income levels, while also confirming reproductive coercion and marital status as significant risk factors. These patterns are well explained by Gender and Power Theory, which emphasizes the role of patriarchal power imbalances in intimate relationships (Connell 1987), and by the Ecological Model of Violence, which situates violence within broader social, cultural, and structural dynamics (Bronfenbrenner 1979). This highlights that violence against women cannot be understood independently of social and cultural contexts, and that status of women and societal dynamics must be considered in efforts to combat violence.

In this study, 18.7% of women experienced reproductive coercion. Prior research reports prevalence rates ranging from 5% in family planning clinics (Upadhyay et al. 2014), 16% among women receiving gynecological care (Clark et al. 2010), to 8% in non-clinical university populations. Our findings are consistent with these data, confirming reproductive coercion as a widespread issue across populations. Factors influencing reproductive coercion include the education levels of women and their spouses, place of residence, income, alcohol use, pregnancy status, decision to continue pregnancy, partner’s refusal to use contraception, and pregnancies desired by only one partner. Our analysis showed that spouse’s education, residence, violence scores, and pregnancy status explained 18.5% of the variance (*p* < 0.001). The model demonstrated a strong negative association with reproductive coercion, with the spouse’s education showing a small but significant negative effect. Violence scores were positively associated with reproductive coercion, while residence and pregnancy status were not significant. These results align with prior studies emphasizing the role of socio-demographic factors and violence in reproductive coercion. Reproductive coercion is a distinct form of abuse involving tactics to control a partner’s reproductive choices (Thaller and Messing 2014). It often co-occurs with physical and sexual violence but can also operate independently through non-violent mechanisms (Grace and Anderson 2018). Behaviors include forcing pregnancy, coercing continuation or termination of pregnancy, and manipulating contraception (Grace and Anderson 2018). Limited evidence suggests that male-dominated fertility ideologies, relationship distrust, and ongoing sexual relationships contribute to reproductive coercion (Thaller and Messing 2014). These findings also reflect the emerging perspective of Gender and Power Theory and Ecological Models, which emphasize how gendered power imbalances in intimate relationships and male entitlement ideology can lead to reproductive control and violence (Heise 1998). Integrating these frameworks highlights the need for theory-driven and culturally sensitive interventions. While the evidence for the impact of reproductive coercion on unplanned pregnancies and contraceptive use was increasing, reproductive coercion appeared as a key aspect of gender-based violence that could be evaluated and considered. As a critical aspect of gender-based violence, understanding its determinants is essential for targeted interventions. Thus, our findings support the need for comprehensive attention to reproductive coercion in both research and clinical settings.

A key finding of this study was the significant associations between unplanned pregnancies and both reproductive coercion (*p* < 0.05) and intimate partner violence (*p* < 0.001) scores. Both relationships demonstrated moderate effect sizes, indicating that unplanned pregnancies are meaningfully related to increased levels of reproductive coercion and violence. Previous research has suggested that IPV contributes to unplanned pregnancy through compromised sexual negotiation, fear of conflict, and limited contraceptive autonomy (Grace and Anderson 2018). Reproductive coercion can be both a precursor and a secondary form of abuse, serving as a mechanism that connects IPV with unplanned pregnancy (Pallitto and O’Campo 2004; Sutherland et al. 2015). The bidirectional and complex relationship between violence and unplanned pregnancy means that violence can increase the risk of unplanned pregnancy, and conversely, unplanned pregnancy may exacerbate violence (Pallitto & O’Campo, 2004). In our study, 41.5% of women who experienced violence also faced reproductive coercion, while 38% of women subjected to reproductive coercion reported violence, supporting the close association between these factors. Women exposed to violence often have limited autonomy over contraceptive use, which may contribute to increased rates of unplanned pregnancy. Notably, 14.5% of participants reported being told by their partner not to use birth control, and 3.8% had been forced to have unprotected sex to become pregnant, highlighting the presence of reproductive coercion within intimate relationships (Miller et al. 2010). Women subjected to violence may also feel afraid to request condom use, further limiting their ability to negotiate contraceptive methods (Grace and Anderson 2018). As a result, they face restricted decision-making power regarding family planning and reproductive choices. Limited control over reproductive health emerges as a critical underlying factor contributing to the heightened risk of unplanned pregnancy among women experiencing abuse (Silverman et al. 2011). Consistent with our results studies, previous findings have indicated that women physically or emotionally abusive relationships are more likely to have unprotected sex, and gender and power, constraint reproductive choices and likely contribute to non-preferred contraceptive use which can be predicted through frameworks such as the Gender and Power Theory and the Ecological Model of Violence (Rosenbaum et al. 2016; Burke and Lindberg 2024). This finding makes an important contribution to the literature, emphasizing the urgent need for integrated interventions that address intimate partner violence, reproductive coercion, and family planning to protect women’s reproductive health and rights. Future research examining the temporal relationship between reproductive coercion and other forms of intimate partner violence may provide valuable insights to strengthen prevention and intervention strategies (Munöz et al.,^2023^). These findings highlight the importance of systematically integrating RC assessments into family planning and gynecology/obstetrics clinics through concise screening tools when RC or violence against women is suspected. Furthermore, incorporating domestic violence screening instruments into intervention designs and increasing healthcare professionals’ awareness of the interconnected and compounding effects of these two forms of gender-based violence are essential steps forward.

Another critical observation was the regional disparity in IPV, reproductive coercion, and unplanned pregnancy. Participants residing in the Eastern Anatolia region reported significantly higher levels of violence and reproductive coercion compared to other regions (*p* < 0.001). These results highlight the influence of sociocultural and socioeconomic factors on reproductive autonomy. Previous studies have shown that women living in patriarchal communities with limited female autonomy and high levels of IPV are more likely to experience reproductive control and unintended pregnancies (Pallitto & O’Campo, 2004; Nikolajski et al. 2015). In Türkiye, national surveys also indicate that violence rates vary regionally, with Eastern and Northeastern Anatolia consistently reporting higher prevalence (Hacettepe University Institute of Population Studies 2015). The current study not only supports these findings but also contributes new data by quantifying the association between regional context and reproductive coercion among pregnant women. These insights underline the necessity of tailoring public health strategies to regional realities, considering local cultural dynamics and gender norms to effectively reduce reproductive coercion and protect women’s reproductive rights. As outlined in the Ecological Model of Violence, these regional and contextual differences again reveal the impact of the interaction between individual, relational, societal and community factors in shaping experiences of violence and coercion (Bronfenbrenner 1979). However, racial and ethnic disparities in reproductive coercion should not be attributed to inherent cultural traits, but rather to structural factors such as economic inequality, systemic racism, and discrimination, which increase vulnerability to such violence (Munoz et al., 2023). By employing these theoretical frameworks, the present study not only reaffirms established risk factors but also illustrates how multi-layered power dynamics and socio-cultural structures shape reproductive autonomy. These findings emphasize the importance of theory-driven and culturally sensitive interventions that address violence across multiple levels, ranging from individual empowerment to broader structural and cultural transformations. In this context, the need for extensive, cross-cultural research involving diverse populations and regional settings is evident. Future studies should also prioritize the development of culturally appropriate counseling interventions to effectively address the multifaceted nature of reproductive coercion.

This study has several limitations. A major limitation is the restricted geographic scope, as data were collected from only two regions/cities, resulting in a less diverse sample. Most participants had primary-level education; therefore, the findings may not be generalizable to women from other regions, those with higher educational attainment, or individuals living in different sociocultural contexts. Future research should include a more diverse and representative sample to better understand women’s experiences of violence and reproductive coercion. Another limitation is the potential for bias due to reliance on self-reported data collected in a hospital setting. Finally, the cross-sectional design, with data collected at a single point in time, precludes any conclusions regarding the directionality or causality of the observed associations.

## Conclusion

In conclusion, this study supported the findings of previous studies. The study revealed the presence of reproductive coercion among women during their reproductive years in our country, demonstrated a significant relationship between violence against women and reproductive coercion, and the highlighted the substantial influence of regional and cultural differences on both reproductive coercion and violence. Our findings also contributed to the existing knowledge on reproductive coercion by describing such coercion in a sample of pregnant women. It was emphasized that the relationship between pregnancy, reproductive coercion, and violence could be studied in similar studies.

The findings of this study highlight the complex relationship between reproductive coercion and pregnancy outcomes. The results indicate the need for further comprehensive research to better understand whether partner coercion leads women to avoid contraceptive methods, thereby increasing the risk of unintended pregnancy, or if pregnancies occur as a direct result of pressure from spouses. Clarifying these mechanisms is crucial for developing effective interventions and policies aimed at protecting women’s reproductive autonomy. Additionally, considering cultural and regional differences will support the creation of context-specific strategies to effectively address reproductive coercion and associated violence.
